# Adult Still’s disease and squamous cell carcinoma in a 69-year-old woman

**DOI:** 10.11604/pamj.2019.34.17.17901

**Published:** 2019-09-09

**Authors:** Sameh Marzouk, Olfa Frikha, Mouna Guermazi, Mouna Snoussi, Moez Jallouli, Zouhir Bahloul

**Affiliations:** 1 Department of Internal Medicine, Hedi Chaker Hospital, Sfax, Tunisia

**Keywords:** Adult-onset Still’s disease, paraneoplastic syndrome, squamous cell carcinoma

## Abstract

Adult-onset Still's disease (AOSD) has been recognized as a cause of fevers of unknown origin. Malignancies are the most important differential diagnoses of AOSD which has been rarely reported in association with cancer. The present paper undertakes the study of a 69-year-old Tunisian woman with AOSD according to the diagnostic criteria of Yamaguchi. She was treated by prednisone, then associated with methotrexate. 18 months later, she developed a squamous cell carcinoma treated with chemotherapy and radiotherapy.

## Introduction

Adult-onset Still's disease (AOSD) is a multisystemic inflammatory disease of unknown etiology. There are no hallmark clinical or pathological findings and, therefore, its diagnosis is based on a set of criteria considered after having ruled out any infection, malignancy or systemic diseases [1]. It is usually encountered in 16 to 35 year old persons and underdiagnosed in the elderly people [2]. We present the case of a patient aged over 65 years who developed a squamous cell carcinoma 18 months after an AOSD onset.

## Patient and observation

A 69-year-old Tunisian female was admitted in 2005 for arthritis and fever. The examination revealed a temperature of 39.7°C, an evanescent diffuse maculopapular rash and symmetrical polyarthritis involving knees, wrists and metacarpophalangeal joints. There was neither lymphadenopathy nor hepatosplenomegaly. Laboratory evaluation revealed: Hb: 74g/l, a white blood cell count of 17.6×10^9^/l (neutrophils: 89%) platelets: 471×10^9^/l, erythrocyte sedimentation rate of 145mm/h and C-reactive protein: 440mg/l. The liver function test scores were elevated: AST: 56 IU/l, ALT: 53IU/l (normal value: 10 to 40IU/l), GGT: 124IU/l (normal value: 10-35IU/l). The serum ferritin was elevated: 3000ng/ml (normal value: 11 to 250ng/ml). Urinalysis was normal and proteinuria was absent. Serological evaluation for hepatitis B and C, Epstein-Barr Virus (EBV), Cytomegalovirus, Herpes simplex, Chlamydia, Mycoplasma, Rickettsia conorii, Coxiella burnetti, Toxoplasma, Borrelia, Aspergillus, Candida, agglutination test for Brucella were negative. Blood, urine and stool cultures were also negative. Tuberculosis was discarded by acid-fast stained smears and culture. Laboratory evaluation for systemic or malignant diseases were negative (antinuclear antibodies, anti-DNA antibodies, antiphospholipid antibodies, anti mitochondrial antibodies, anti smooth-muscle antibodies, rheumatoid factor, Antineutrophil Cytoplasmic Antibodies (ANCA), cryoglobulins, tumor markers). Chest x-rays, ultrasound examination, and echocardiography were normal as well. The temporal artery biopsy and the bone marrow examination were normal. We diagnosed AOSD according to the Yamaguchi criteria [1] and we started a steroid therapy (prednisone: 1mg/kg/day). The symptoms began to improve. Ten months later, she presented the same clinical picture. X-rays of both hands showed bone erosions at the carps. We used oral methotrexate in weekly dose of 10mg. 8 months later, the patient developed an ulcerated tumor at the scalp with cervical lymph nodes whose biopsy revealed a squamous cell carcinoma. Methotrexate was stopped and she was treated with chemotherapy and radiotherapy. She did not present a recurrence during a 12 months follow up period.

## Discussion

Adult Still's disease is a systemic disease of unknown origin without any pathognomonic signs. It is one of the febrile disorders of unknown etiology, typically characterized by a spiking fever with an evanescent rash and the involvement of various organs [1]. Recently, glycosylated ferritin has been reported to be helpful in diagnosing AOSD [3]. Because this disease almost lacks specific clinical, laboratory, and histological features, the exclusions of infections, malignancies, and other rheumatic diseases are an important diagnostic procedure [4]. Among the differential diagnosis of AOSD, it is important to rule out malignant diseases like Hodgkin disease or non-Hodgkin lymphoma, solid cancers (kidney, colon, and lung), myeloproliferative disorders, and paraneoplastic syndrome [5]. It is worthy to note that few cases of malignancy associated with AOSD are reported in the literature. Besides, the relationship between haematological malignancies (lymphoma and leukemia) and AOSD has been widely reported. However, publications pertaining to the possible relationship between neoplasia and AOSD are rare [6]. In fact, Cabane *et al.* [7] reported an AOSD associated with a larynx carcinoma. Furthermore, Ahn *et al.* [8] accounted for an AOSD diagnosed concomitantly with occult papillary thyroid cancer. Shibuya [9] described an esophageal cancer diagnosed 9 months after the onset of this disease in a 77-year-old man. The present paper presents a new case of AOSD that appears in old women. The diagnosis was retained after the exclusion of infections, malignancies and rheumatic diseases. The carcinoma has emerged after 18 months of evolution, while the AOSD is in remission, eliminating the assumption that it is a paraneoplastic syndrome.

## Conclusion

Although adult Still's disease most commonly affects young adults, it is proven to be a disease that can affect all categories of age. We undertake the study of an old AOSD female patient who was treated with Methotrexate. In her case, the occurrence of squamous cell carcinoma is considered as an association with AOSD.

## Competing interests

The authors declare no competing interests.
